# Clustering in dilated cardiomyopathy at initial evaluation: An effective tool for clinical stratification

**DOI:** 10.1002/ejhf.3780

**Published:** 2025-08-15

**Authors:** Ilaria Gandin, Maria Perotto, Alessia Paldino, Giovanni Baj, Denise Zaffalon, Andrea Pezzato, Cinzia Crescenzi, Fabiana Romeo, Annamaria Martino, Francesca Fanisio, Federica Toto, Maddalena Rossi, Marta Gigli, Matteo Dal Ferro, Leonardo Calò, Gianfranco Sinagra, Marco Merlo

**Affiliations:** ^1^ Biostatistics Unit, Department of Medicine, Surgery and Health Science University of Trieste Trieste Italy; ^2^ Cardiovascular Department, Azienda Sanitaria Universitaria Giuliano Isontina (ASUGI) University of Trieste Trieste Italy; ^3^ Department of Mathematics, Informatics and Geosciences University of Trieste Trieste Italy; ^4^ Cardiology Department, Azienda Unità Locale Socio‐Sanitaria n. 2 Marca Trevigiana Montebelluna Italy; ^5^ Division of Cardiology, Policlinico Casilino Rome Italy

**Keywords:** Dilated cardiomyopathy, ECG, Clustering analysis, Machine learning, Arrhythmic risk

## Abstract

**Aims:**

Dilated cardiomyopathy (DCM) has a highly variable presentation and disease course. Current stratification strategies are complex and require multimodality evaluation. Using machine learning (ML) on a large dataset obtained at first cardiological evaluation, this study aims to identify specific DCM subgroups.

**Methods and results:**

In a retrospective cohort of DCM patients, baseline clinical, genetic, and outcome data were collected. Unsupervised clustering was performed and then simplified to identify patient subgroups. The subgroups were characterized based on outcomes, including all‐cause mortality/heart transplantation (HT)/left ventricular assist device implantation (LVAD), sudden cardiac death/major ventricular arrhythmias (SCD/MVA) and heart failure‐related death/HT/LVAD. These findings were then validated in an external population. In the derivation cohort of 409 patients (mean age 46 ± 14 years, 71% male), two cluster‐subgroups were identified: CL1 (82%) and CL2 (18%), mainly differentiated by electrocardiogram (ECG) characteristics. A lower yield of pathogenic/likely pathogenic variants was found in CL2 versus CL1 (15% vs. 47%, *p* < 0.001). A simplified clustering using only three variables (QRS duration, presence of left bundle branch block, intrinsicoid deflection >50 ms) was equally effective and validated in the external cohort of 160 patients (mean age 54 ± 13 years, 68% male). A lower risk for SCD/MVA events was observed for CL2 in the primary (hazard ratio 0.29, 95% confidence interval 0.13–0.67) and validation cohort (*p* = 0.017).

**Conclusions:**

Using ML, baseline ECG variables were found to effectively identify two DCM subgroups differing in disease progression and genetic background. This approach could serve as a valuable tool for improving risk stratification of DCM patients upon their initial evaluation.

## Introduction

Dilated cardiomyopathy (DCM) is a heterogeneous disease in terms of clinical presentation and genetic background. Although multiple phenotypic and genetic findings have been suggested as possible prognostic features of heart failure (HF) and severe arrhythmic events,^1^, ^2^, ^3^ no definite prognostic risk score has yet been validated in this field.^4^

Artificial intelligence (AI) and machine learning (ML) have proven helpful in identifying distinct phenogroups within the DCM spectrum, each associated with a unique clinical presentation, distinct underlying aetiology and outcomes.^5^, ^6^ However, this approach required the collection of advanced information, including second and third level exams, such as cardiac magnetic resonance (CMR) data and even endomyocardial biopsy. Similar results were obtained from cardiac transcriptome of patients affected by DCM.^7^ While these studies demonstrate common features shared by many DCMs, the input data required for the clustering algorithms is often not available upon first medical contact, not always collected during follow‐up, and therefore not applicable in all cardiac centres.

Electrocardiogram (ECG) remains the simplest, most reliable, and lowest‐cost technology with excellent diagnostic and prognostic functions.^8^ The recently published Madrid Genotype Score, which estimates the likelihood of a positive genetic test in DCM, incorporates two ECG parameters among its five criteria—low voltage on the ECG and absence of left bundle branch block (LBBB)—underscoring the clinical importance of ECG.^9^

Leveraging the potential of ML in stratifying patient prognosis in DCM only with data that can be collected at initial evaluation, the aims of our study were: (i) to apply unsupervised clustering to patients affected by DCM using clinical information available at first medical contact; (ii) to provide a characterization of clustered patients based on phenotypic similarities; and (iii) to analyse these clusters in terms of outcomes and genetic background.

## Methods

### Study cohort and study design

The present observational retrospective study includes a primary cohort of patients affected by DCM who underwent genetic testing, recruited from the Familial Cardiomyopathy Registry of Trieste (Italy).^1^, ^10^ DCM was defined as left ventricular dilatation (left ventricular end‐diastolic diameter >58 mm in males, and >52 mm in females^11^) and impaired ejection fraction (left ventricular ejection fraction [LVEF] <50%), following a thorough exclusion of secondary causative aetiologies.^11^, ^12^ Of note, non‐dilated left ventricular cardiomyopathy (NDLVC) with only systolic dysfunction (i.e. LVEF <50% at enrolment) was included.

All patients underwent a baseline evaluation upon study enrolment. Collected information included demographic and clinical data (inclusive of HF symptoms and New York Heart Association [NYHA] functional class), information on family history of cardiomyopathy and familial sudden cardiac death (SCD), results of 12‐lead ECG and 24‐h Holter ECG monitoring, and echocardiographic assessment. Transthoracic echocardiogram with biventricular dimensions and systolic function was assessed according to international guidelines.^13^

Regarding ECG, a wide collection of features was recorded (online supplementary *Table* *S1*). Results of genetic testing, endomyocardial biopsy, CMR and cardiac exercise stress testing information were not included in the clustering dataset as they are often unavailable at the first cardiological evaluation.

The study included a validation cohort of DCM patients, both genotyped and not, enrolled in the CARITMO (Cardiopatie ARITMOgene) registry of the Casilino Hospital in Rome (Italy).

The study was approved by the ethics committee (CEUR N.O. 43/2009, Em 06/22).

### Genetic testing

All enrollees were screened for genetic variants associated with DCM by next generation sequencing of multigene panels, as previously reported.^1^, ^10^, ^14^ Variants were classified as pathogenic or likely pathogenic (P/LP) according to the American College of Medical Genetics and Genomics criteria.^15^

### Endpoints

There were three composite endpoints: (1) all‐cause mortality or heart transplantation (D/HT); (2) severe arrhythmic events including SCD and major ventricular arrhythmias (SCD/MVA); and (3) HF‐related events including HF death, HT and left ventricular assist device implantation (HF/HT). SCD included witnessed SCD with or without documented ventricular fibrillation (VF), death within 1 h of acute symptoms, or nocturnal death with no antecedent history of immediate worsening symptoms. MVA was defined as VF, sustained ventricular tachycardia (lasting >30 s or with haemodynamic instability), and appropriate ICD interventions (shock or anti‐tachycardia pacing on VF or sustained ventricular tachycardia).

### Statistical analysis

#### Clustering

Given the framework of the study, which is based on a data‐driven approach, the principle followed in the data collection for clustering analysis was to retain as much information as possible. This resulted in a large set of covariates that reflect the result of the above‐mentioned tests, with high levels of correlations. Covariates were retained if the missing rate was lower than 10%. Binary variables with lower frequency class <2% were also excluded. A full list of the features included in the analysis can be found in online supplementary *Table* *S1*. The analysis presented two major challenges: the large number of variables and their mixed‐data type (presence of both numerical and categorical variables). For this reason, a two‐step approach was followed. First, a dimensionality reduction technique called PCAmix,^16^ which extends the principal component analysis to mixed data, was applied (see online Supplementary Information). Secondly, a clustering method was used on the reduced dataset formed by 11 principal components. More specifically, an agglomerative hierarchical clustering algorithm was applied. The optimal number of clusters was identified using the average silhouette criteria.^17^

Genetic information was not part of the input features of the cluster analysis since the focus of the present clustering was a phenotypic characterization with elements the clinician has available upon first medical evaluation. Instead, the presence of P/LP variants was investigated in relationship with cluster partition to understand possible differences between clusters in terms of disease aetiology. For this purpose, genes *DSP*, *PKP2*, *FLNC*, and *LMNA* were grouped as ‘arrhythmic genes’ and all the remaining as ‘non‐arrhythmic genes’.

#### Simplified clustering and validation

To provide a clustering rule that can be easily applied in clinical practice, and to the external cohort of the present study, a simplified version of the clustering model, involving only a reasonable number of variables, was obtained using a penalized logistic regression. A LASSO model (a standard technique for variable selection problems^18^) was estimated in the study cohort with all the input variables involved in the clustering as predictors, and the cluster assignment as outcome. The penalty parameter was selected such that only three predictors were not shrunk to zero. The performance of such simplified model was evaluated in terms of receiver‐operating characteristic area under the curve (AUC) through a 10‐fold cross‐validation. Based on the continuous score of the simplified model (ranging from 0 to 1), individuals were assigned to cluster 2 if exceeding the value 0.23, corresponding to the Youden's cut‐point.

#### Group comparison

Continuous variables were compared between groups using the *t*‐test or the non‐parametric Mann–Whitney U test as appropriate. Discrete variables were analysed using the chi‐square or Fisher's exact test. For the D/HT endpoint, Kaplan–Meier survival curves were estimated and compared between groups with the log‐rank test. For the SCD/MVA endpoint, cumulative incidence curves at 20 years of maximum follow‐up were obtained considering D/HT and HF/HT competing events and compared with Gray's test. The same approach was followed for the HF/HT endpoint, for which SCD/MVA and D/HT were competing events. To include the effect of known risk factors, multivariate cause‐specific Cox models were obtained and nested models were compared using the likelihood ratio test and Harrell's C‐index. Martingale residual plots were visually inspected to assess linearity. Statistical analyses were performed in R (version 4.4.1) using the packages ‘PCAmixdata’, ‘cluster’, ‘survival’, ‘cmprisk’. A *p*‐value <0.05 was considered statistically significant.

## Results

### Derivation cohort and clustering

The derivation cohort consisted of 409 patients affected by DCM. The majority were men (*n* = 291, 71%) and mean age at time of recruitment was 46 years (standard deviation [SD] 14). *Table* *1* reports main demographic and clinical characteristics at baseline of the derivation cohort (additional data in online supplementary *Table* *S2*).

**Table 1:** Baseline characteristics of the study cohort and the external validation cohort

	Study cohort (*n* = 409)	Validation cohort (*n* = 160)	*p*‐value
Age, years, mean (SD)	46 (14)	54 (13)	<0.001
Men, *n* (%)	291 (71)	108 (68)	0.4
European, *n* (%)	408 (99)	160 (100)	0.9
Genetic positive P/LP variants, *n* (%)	169 (41)	33 (39), 76 not tested	0.8
Arrhythmic gene P/LP variants, *n* (%)	53 (31)	14 (42)	0.3
LVEF, %, mean (SD)	35 (12)^a^	38 (10)	
LV end‐diastolic diameter, mm, mean (SD)	63 (10)^b^	60 (7)	

LV, left ventricular; LVEF, left ventricular ejection fraction; P/LP, pathogenic/likely pathogenic; SD, standard deviation.

^a^ Missing data in one individual.

^b^ Missing data in five individuals.

**Figure 1:**
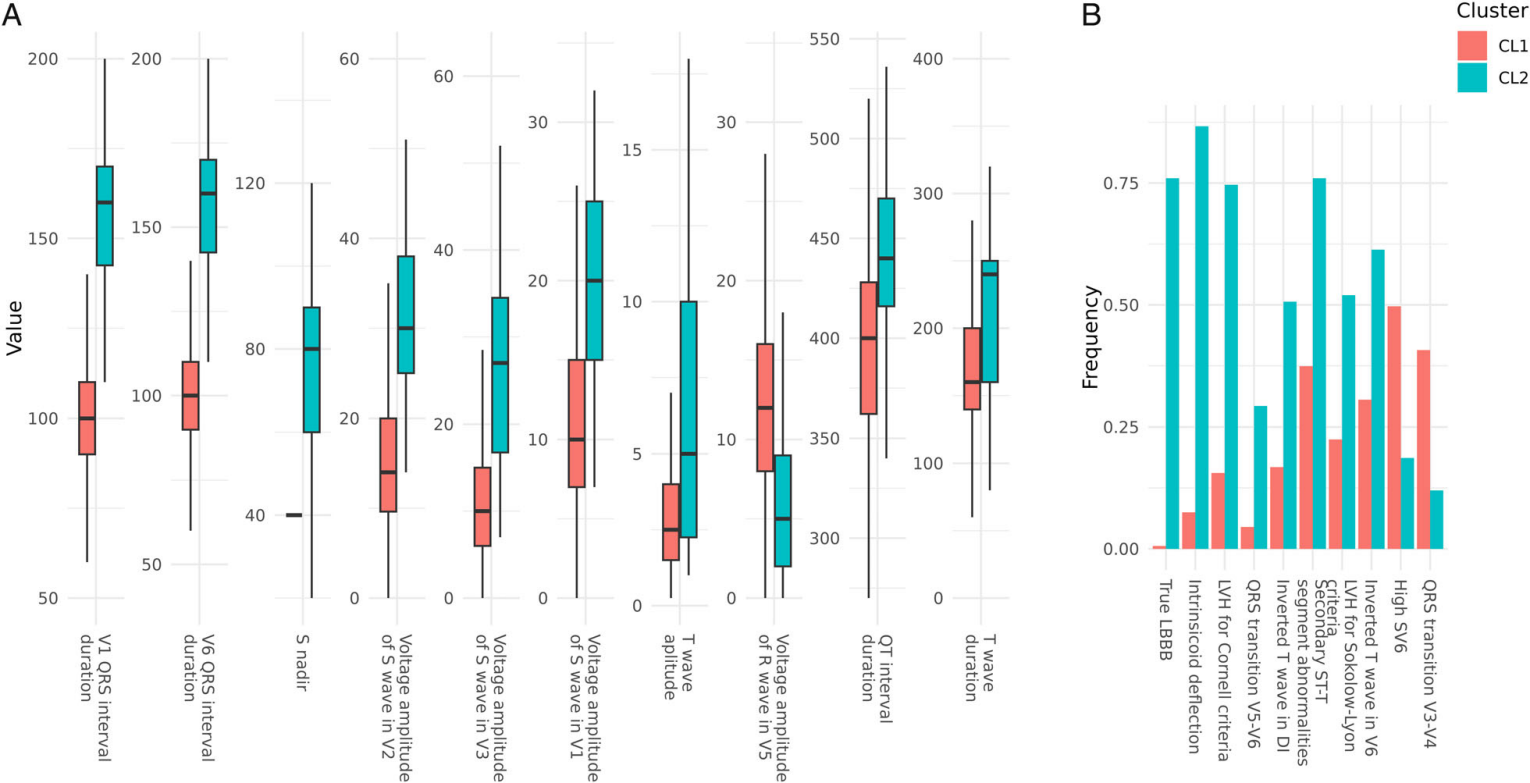
Distinctive features of the two clusters. First 10 continuous variables ordered by mean difference after standardization (*A*) and first 10 binary variables ordered by standardized chi‐square residuals (*B*) are shown to provide a description of the cluster composition.

**Figure 2:**
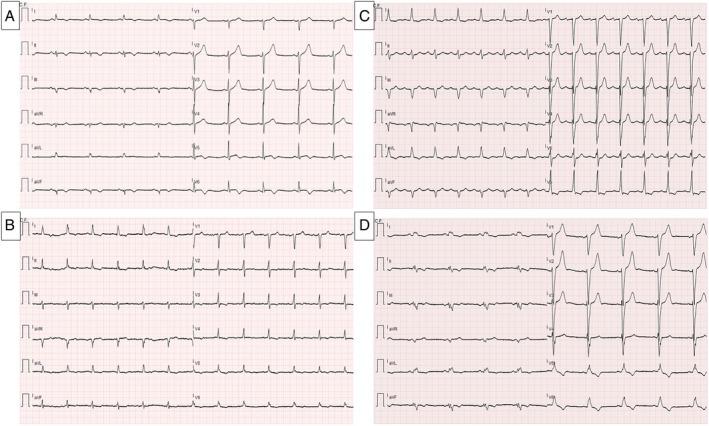
Patients' electrocardiograms. Examples of electrocardiograms of patients belonging to cluster 1 (*A*, *B*) and cluster 2 (*C*, *D*).

A total of 102 input features were initially considered for the cluster analysis. Following the PCAmix strategy, we obtained a dataset with reduced dimensionality preserving 47% of the variance, which were fed to the hierarchical clustering algorithm. This clustering was able to recognize two main DCM clusters: a first cluster (CL1) of 334 individuals (82%) and a second cluster (CL2) of 75 individuals (18%). Considering characteristics at baseline (*Table* *2*), compared to CL1, subjects of CL2 showed a less arrhythmic profile (5% vs. 20% of premature ventricular contractions/24 h, *p* = 0.002, 28% vs. 43% of non‐sustained ventricular tachycardia, *p* = 0.020) and a worse LVEF (median value 30% vs. 35%, *p* = 0.005). Features that most differentiated the two groups were mainly ECG parameters (*Figures* *1* and *2*). Compared to CL1, the CL2 group had a higher prevalence of true LBBB (76% vs. 1%, *p* < 0.001), intrinsicoid deflection in V5 or V6 (87% vs. 7%, *p* < 0.001), and left ventricular hypertrophy (LVH) by Cornell criteria (75% vs. 16%, *p* < 0.001), together with higher QRS duration in V1 (median value 160 vs. 100 ms, *p* < 0.001), higher QRS duration in V6 (median value 160 vs. 100 ms, *p* < 0.001) S wave voltage (in V1 or V2, median value 30 vs. 14 mm, *p* < 0.001), and S wave duration in V1 or V2 (S nadir‐to‐end, median value 80 vs. 40 ms, *p* < 0.001).

**Table 2:** Comparison of baseline characteristics between cluster 1 and cluster 2.

	CL1 (*n* = 334)	CL2 (*n* = 75)	*p*‐value
DCM clinical characteristic			
Age, years, mean (SD)	45 (14)	50 (10)	0.001
Men, *n* (%)	251 (75)	40 (53)	<0.001
Genetic positive, *n* (%)	158 (47)	11 (15)	<0.001
Premature ventricular contractions/24 h, *n* (%)	68 (20)	4 (5)	0.002
Non‐sustained ventricular tachycardia, *n* (%)	142 (43%)	21 (28%)	0.020
LVEF, %, median (IQR)	35 (26–45)	30 (23–38)	0.005
LVEF <35%, *n* (%)	165 (49)	51 (68)	0.004
LV end‐diastolic diameter, mm, median (IQR)	62 (57–68)	62 (58–72)	0.5
Atrial fibrillation, *n* (%)	14 (4.2%)	0 (0%)	0.083
Cluster distinctive features			
True LBBB, *n* (%)	2 (1)	57 (76)	<0.001
Intrinsicoid deflection in V5 or V6, *n* (%)	25 (7)	65 (87)	<0.001
LVH by Cornell criteria, *n* (%)	52 (16)	56 (75)	<0.001
QRS duration in V1, ms, median (IQR)	100 (90–110)	160 (142–170)	<0.001
QRS duration in V6, ms, median (IQR)	100 (90–110)	160 (142–170)	<0.001
S nadir to end in V1 or V2, ms, median (IQR)	40 (40–40)	80 (60–90)	<0.001
S wave amplitude in V1 or V2, mm, median (IQR)	14 (10–20)	30 (25–38)	<0.001

CL1, cluster 1; CL2, cluster 2; DCM, dilated cardiomyopathy; IQR, interquartile range; LBBB, left bundle branch block; LV, left ventricular; LVEF, left ventricular ejection fraction; LVH, left ventricular hypertrophy; SD, standard deviation.

### Clinical outcomes in phenotypic groups

**Figure 3:**
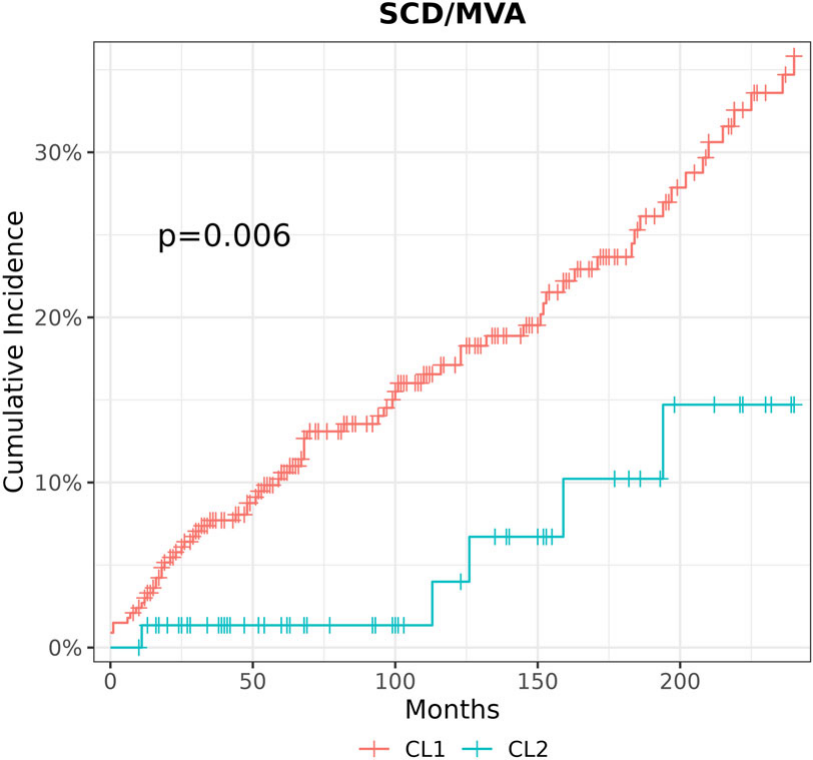
Risk of severe arrhythmic events in the study cohort. Comparison of cumulative incidences for sudden cardiac death/major ventricular arrhythmia (SCD/MVA) events between cluster 1 (CL1) and cluster 2 (CL2) in the study cohort.

During a median follow‐up of 100 months (interquartile range [IQR] 51–185), 66 individuals met the primary endpoint (D/HT), while 92 SCD/MVA events and 46 HF events were recorded. For the primary endpoint and the HF‐related endpoint, there were no differences between CL1 and CL2 (online supplementary *Figures* *S1* and *S2*). Conversely, a lower risk for SCD/MVA events was observed in CL2 (*p* = 0.006) (*Figure* *3*) with an associated hazard ratio of 0.29 (95% confidence interval [CI] 0.13–0.67). When considering the effect of sex, age, family history of SCD, lower lateral T waves, syncope, NYHA class III or IV, LVEF, the inclusion of CL2 information had a significant impact (adjusted hazard ratio 0.20, 95% CI 0.08–0.48, *p* < 0.001) and increased C‐index from 0.69 to 0.74 (*Table* *3*). Given the higher prevalence of true LBBB in CL2, the role of cardiac resynchronization therapy (CRT) was investigated to determine whether CRT had a role in CL2 prognosis. Considering a prognostic model including CRT presence, the inclusion of CL2 information improved the model (*p* = 0.0002) and increased C‐index from 0.68 to 0.74. The association between clusters and SCD/MVA events was also investigated after excluding individuals with NDLVC (*n* = 82, 20%), yielding similar results (adjusted hazard ratio 0.15, 95% CI 0.05–0.44, *p* < 0.001) (online supplementary *Table* *S3*).

**Table 3:** Multivariable analysis for sudden cardiac death/major ventricular arrhythmia events including cluster 2

Characteristic	HR	95% CI	*p*‐value
Sex	1.56	0.90–2.73	0.12
Age	1.02	1.00–1.04	0.069
Family history of SCD	1.33	0.73–2.40	0.3
Inverted T wave in infero‐lateral leads	1.54	0.89–2.68	0.12
Syncope	4.14	2.35–7.30	<0.001
NYHA class III‐IV	1.54	0.87–2.70	0.14
LVEF	0.98	0.96–1.01	0.2
CL2	0.20	0.08–0.48	<0.001

CI, confidence interval; CL, cluster 2; HR, hazard ratio; LVEF, left ventricular ejection fraction; NYHA, New York Heart Association; SCD, sudden cardiac death.

### Genetic yield in phenotypic groups

The relationship between the genetic aetiology of the disease and the two clusters was analysed. A lower yield of P/LP variants was found in CL2 compared to CL1 (15% vs. 47%, *p* < 0.001). Given the association found between CL2 and a lower risk for SCD/MVA events, the rate of P/LP variants in arrhythmic genes (*DSP*, *PKP2*, *LMNA*) was compared: CL1 included 20 cases (6%) and CL2 none (0%, *p* = 0.033).

### Validation in external cohort

Using a penalized logistic model, we obtained a simpler clustering rule that was able to classify individuals in CL1 and CL2 using only three variables, maintaining a high accuracy (AUC = 0.991, standard error = 0.005). Variables involved were QRS duration in V6, true LBBB, and intrinsicoid deflection >50 ms (online supplementary *Table* *S4*).

This simplified model was applied to the validation cohort, which included 160 patients, 108 men (68%) with a mean age at baseline of 54 years (SD 13) (*Table* *1*). In the validation cohort, 126 individuals (79%) were assigned to CL1 and 34 individuals (21%) to CL2.

During a median follow‐up of 50 months (IQR 25–84), 3 patients died, and 21 patients experienced an SCD/MVA event (no HF events were recorded). There was no significant association between clusters and D/HT events (*p* = 0.3). Instead, CL2 was associated with a lower risk of SCD/MVAs (*p* = 0.017) (online supplementary *Figure* *S3*). More specifically, none of the individuals belonging to CL2 experienced SCD/MVA events.

## Discussion

With this study, we aimed to explore the potential of clinical information collected at the very first evaluation (ECG, echocardiographic, Holter ECG and clinical data) in recognizing distinct high‐risk subgroups of patients with a diagnosis of DCM or NDLVC with systolic dysfunction by the application of an unsupervised clustering methodology.

In this study, two clusters were identified, which differed mainly in terms of ECG presentation, as depicted in the *Graphical Abstract*. The two groups showed an interesting antithetical genetic background and incidence of arrhythmic outcomes during follow‐up. These clusters were associated with higher risk for arrhythmic outcomes even when considered alongside commonly recognized risk factors for MVA, such as LVEF or family history of SCD. For a future clinical application, we were able to simplify clustering variables to only three of them, maintaining high accuracy in distinguishing clusters, and showing reproducibility in an external validation cohort from another centre.

This is the first study showing how ECG data, easily available at first evaluation, can effectively cluster patients affected by DCM. This is particularly of interest when considering that other types of information were fed into the algorithm, such as echocardiographic data, but a cardiomyopathy‐oriented ECG interpretation was selected by the algorithm to differentiate the two clusters.

If CL1 exhibited high variability in terms of clinical findings, genetic background and outcomes, CL2 included subjects with more uniform characteristics. CL2 is indeed the smallest and most consistent group in which genetic analysis more often yielded negative results, and ECG parameters more frequently indicated increased QRS duration, LBBB, and markers of delayed and slower ventricular depolarization. This CL2 showed very few arrhythmic events during an 8‐year follow‐up and was, in fact, devoid of P/LP variants of well‐known arrhythmic genes such as *DSP*, *PKP2*, and *LMNA*. This held true even if more than half of the patients in CL2 had LVEF <35%.

It is of interest that the main ECG clustering variables identified, all pertained to the same sphere of indicators of left ventricular remodelling (i.e. hypertrophy/dilatation): patients in CL2 significantly more often met LVH Cornell criteria, had more delayed intrinsicoid deflection, more often LBBB and wider QRS. It could be hypothesized that a larger left ventricular mass (hypertrophy criteria, increased time for the electric signal to go from the endocardium to the epicardium) and fewer indicators of myocardial fibrosis (high voltages) in the field of DCM might protect from major arrhythmias through a less compromised myocardial muscle.

On one hand, our results are in line with the recently published Madrid Genotype Score which proved to be an accurate tool to predict genetic test positivity in DCM.^9^ In fact, the absence of LBBB and low voltages are predictors of a positive genetic test in this score. Complementarily, we found that high voltages and LBBB identify a cluster of gene‐elusive DCMs. On the other hand, in a previous analysis of our group focused on ECG in DCM population,^19^ the only presence of true LBBB did not show a prognostic role in terms of HF and arrhythmic events. However, true LBBB is not the only parameter representing CL2, and other CL2 parameters (LVH by Cornell criteria, S nadir, high QRS voltages) were in the same study described as protective for MVA/SCD and LVH also for overall survival. Whether CL2 patients had truly idiopathic DCM could be questioned. Although the diagnosis was properly based on the exclusion of confounding environmental factors, it cannot be excluded that CL2 patients represent a cohort of patients in which the role of these factors is not negligible.

### Clinical implications

Despite a strong prognostic role herein identified, the literature is poor of studies that investigate the role of ECG parameters in DCM and is instead rich in second‐ and third‐line tests in search for deep cardiomyopathy phenotyping. It would be worth reconsidering the wealth of information ECG can provide on DCM patients and further explore its predictive role in future studies, especially since this information is already used in clinical practice.

While ML represents a powerful and innovative approach to multidimensional data problems,^20^, ^21^ it may be often difficult to integrate them in clinical practice firstly because they usually need a wide range of input variables and secondly because their result could be difficult to interpret based on clinical experience alone.^22^, ^23^ With this in mind, we believe the few variables we identified, and their immediate collection, have the merit of being easily applied by all clinicians, without the need for complex calculations. One of the implications of such an easily applied model is that also cardiologists not working in cardiomyopathy referral centres can use it to estimate the priority with which they need to refer a suspected DCM patient to a tertiary cardiomyopathy clinic. DCM is a widely heterogeneous condition, that often challenges the clinician especially upon first patient evaluation when it may be difficult to foresee which path will the disease take. In the future, the findings of this study could be put together with known prognostic models to better stratify patients with DCM from their very first medical contact.

### Limitations

Although sufficient for statistical analysis, the number of patients included in this study is limited and further testing of the model in larger cohorts should be performed. Also, in line with previous literature,^24^, ^25^ the study population included both DCM and NDLVC with systolic dysfunction (a smaller percentage), which, although distinct categories, have been recently demonstrated to carry similar prognosis.^26^ While all results could be entirely replicated in the DCM patients alone, the number of NDLVC patients was too small for that. Further studies will be needed to better characterize this newly defined cardiomyopathy. Furthermore, although validated in an external cohort of DCM (both genotyped and not), the selection bias of deriving the algorithm from patients enrolled in a cardiomyopathy referral centre, only genotyped, cannot be excluded. Compared to CL2, which is smaller and clinically distinct, our largest cluster comprises patients with more heterogeneous characteristics. CL1 may harbour underlying additional substructure, however a larger population is needed to provide a deeper characterization. Nonetheless, the identification of a low‐risk cluster is an important steppingstone for the clinical cardiologist.

## Conclusions

Unsupervised clustering analyses using data collected at the first evaluation of DCM patients robustly identified a low‐risk cohort of patients with distinctive ECG characteristics, possibly expression of a different underlying pathologic process. The implementation of this model with simple ECG parameters in clinical practice will help in identifying from the start patients at lower risk for arrhythmic outcomes.

## Supporting information


**Data S1** Supporting Information.

**Figure S1 Death and heart transplant survival curve**. Kaplan–Meier curve for D/HT events in CL1 and CL2

**Figure S2 Heart failure in the two clusters**. Cumulative incidence functions for HF events in CL1 and CL2.

**Figure S3 Risk of severe arrhythmic event in the validation cohort**. Comparison of Kaplan–Meier curves for SCD/MVA events between CL1 and CL2 in the validation cohort.
