# Supplementary material for: Clustering in dilated cardiomyopathy at initial evaluation: An effective tool for clinical stratification
Source: Eur J Heart Fail. 2025 Aug 15;27(12):3040–7. doi: 10.1002/ejhf.3780 (PMC12803628; doi:10.1002/ejhf.3780)
Supplement: Supplementary file 1 — Data S1 Supporting Information. [file EJHF-27-3040-s004.docx]

**Supplemental material**

**PCAmix**

PCAmix extends principal component analysis (PCA), which is a standard multivariate analysis method for reducing the dimensions in case of a large number of variables per observation, to mixed data datasets. Similarly to PCA, the method uses a generalized singular value decomposition to obtain a geometric transformation and a subspace (by projection) that preserves the information by maximizing the variability of the projected observations. As result, new variables are obtained (“principal components”, PCs) that are linear combinations of the original variables, ordered by maximum dispersion, and by construction non correlated among each other. By considering only the first *p* PCs, the dimension of the dataset is reduced. For this analysis, the first 11 PCs were considered (that is, PCs for which the proportion of explained variance/#PCs>2%).

**Supplemental figure legends**

**Supplemental figure 1**. **Death and heart transplant survival curve.** Kaplan-Meier curve for D/HT events in CL1 and CL2

**Supplemental figure 2.** **Heart failure in the two clusters.** Cumulative incidence functions for HF events in CL1 and CL2.

**Supplemental figure 3**. **Risk of severe arrhythmic event in the validation cohort.** Comparison of Kaplan-Meier curves for SCD/MVA events between CL1 and CL2 in the validation cohort.

**Supplemental tables**

**Supplementary Table S1.** List of input variables

| **Name** | **Domain** | **Type** | **Unit of measurement** | **Description** | **Missing rate (%)** |
| --- | --- | --- | --- | --- | --- |
| Sex |  | binary | male | Sex | 0 |
| Family history of SCD |  | binary |  | Family history of SCD | 1 |
| Age |  | numeric | years | Age | 0 |
| Weight |  | numeric | Kg | Weight | 1 |
| Systolic blood pressure |  | numeric | mmHg | Systolic blood pressure | 1 |
| Syncope |  | binary |  | Syncope | 1 |
| Nonsustained ventricular tachycardia (NSVT) | Holter ECG | binary |  | Nonsustained ventricular tachycardia (NSVT) | 6 |
| Left ventricle ejection fraction (LVEF) | Echocardiogram | numeric | % | Left ventricle ejection fraction (LVEF) | 0 |
| Left ventricle end diastolic diameter (LVEDD) | Echocardiogram | numeric | mm | Left ventricle end diastolic diameter (LVEDD) | 1 |
| Restrictive filling pattern | Echocardiogram | binary |  | Restrictive filling pattern | 2 |
| Left atrial area (LAA) | ECG | numeric | cm2 | Left atrial area (LAA) | 5 |
| Sinus rhytm | ECG | binary |  | Sinus rhytm | 0 |
| Atrial fibrillation | ECG | binary |  | Atrial fibrillation | 0 |
| Cardiac frequency | ECG | numeric | beats per minute | Heart rate | 0 |
| Supraventricular premature beats | Holter ECG | binary |  | Supraventricular premature beats | 0 |
| Ventricular premature beats> 1000 | Holter ECG | binary |  | Ventricular premature beats> 1000 | 0 |
| QRS axis | ECG | numeric | degrees | QRS axis | 0 |
| T wave axis | ECG | numeric | degrees | T wave axis | 0 |
| QRS-T angle | ECG | binary |  | absolute value of difference between QRS- and T-axis yielding values between 0 and 180°, and it was categorized as normal (≤ 90°) or abnormal (≥100°) | 0 |
| First degree AV block | ECG |  | ms | PQ interval ≥200 ms | 2 |
| PQ interval duration | ECG |  | ms | PQ interval duration | 0 |
| V1 QRS interval duration | ECG |  | ms | QRS interval duration in lead V1 | 0 |
| V6 QRS interval duration | ECG |  | ms | QRS interval duration in lead V6 | 0 |
| QT interval duration | ECG |  | ms | QT interval duration in V5, V4 or V6) | 0 |
| Left atrial enlargement | ECG | binary |  | bifidus P wave in DII or DI or aVL; duration > 0.11 ms | 2 |
| Right atrial enlargement | ECG | binary |  | high P wave in DII or DIII or aVF; amplitude > 0.25 mV | 2 |
| Q wave in DI | ECG | binary |  | Q waves (> 40 ms (1 mm) wide, > 2 mm deep, > 25% of depth of QRS complex) | 0 |
| Q wave in DII | ECG | binary |  | Q waves (> 40 ms (1 mm) wide, > 2 mm deep, > 25% of depth of QRS complex) | 0 |
| Q wave in DIII | ECG | binary |  | Q waves (> 40 ms (1 mm) wide, > 2 mm deep, > 25% of depth of QRS complex) | 0 |
| Q wave in aVF | ECG | binary |  | Q waves (> 40 ms (1 mm) wide, > 2 mm deep, > 25% of depth of QRS complex) | 0 |
| Q wave in aVR | ECG | binary |  | Q waves (> 40 ms (1 mm) wide, > 2 mm deep, > 25% of depth of QRS complex) | 0 |
| Q wave in aVL | ECG | binary |  | Q waves (> 40 ms (1 mm) wide, > 2 mm deep, > 25% of depth of QRS complex) | 0 |
| Q wave in V1 | ECG | binary |  | Q waves (> 40 ms (1 mm) wide, > 2 mm deep, > 25% of depth of QRS complex) | 0 |
| Q wave in V2 | ECG | binary |  | Q waves (> 40 ms (1 mm) wide, > 2 mm deep, > 25% of depth of QRS complex) | 0 |
| Q wave in V3 | ECG | binary |  | Q waves (> 40 ms (1 mm) wide, > 2 mm deep, > 25% of depth of QRS complex) | 0 |
| Q wave in V5 | ECG | binary |  | Q waves (> 40 ms (1 mm) wide, > 2 mm deep, > 25% of depth of QRS complex) | 0 |
| Q wave in V6 | ECG | binary |  | Q waves (> 40 ms (1 mm) wide, > 2 mm deep, > 25% of depth of QRS complex) | 0 |
| Q wave in infero-lateral | ECG | binary |  | Q waves (> 40 ms (1 mm) wide, > 2 mm deep, > 25% of depth of QRS complex), infero-lateral | 0 |
| Q wave in antero-lateral | ECG | binary |  | Q waves (> 40 ms (1 mm) wide, > 2 mm deep, > 25% of depth of QRS complex), antero-lateral | 0 |
| True LBBB | ECG | binary |  | QS or rS in V1-V2; duration > 140 ms if men, > 130 ms if women; notch or clurring in > 2 leads DI, aVL, V1,V2,V5,V6 | 0 |
| Complete LBBB | ECG | binary |  | QRS duration greater than or equal to 120 ms in adults, greater than 100 ms in children 4 to 16 years of age, and greater than 90 ms in children less than 4 years of age; Broad notched or slurred R wave in leads I, aVL, V5, and V6 or RS pattern in V5 and V6 attributed to displaced transition of QRS complex | 0 |
| Incomplete LBBB | ECG | binary |  | QRS duration between 110 and 119 ms; Presence of left ventricular hypertrophy pattern; R peak time greater than 60 ms in leads V4, V5, and V6; Absence of q wave in leads I, V5, and V6 | 0 |
| Complete RBBB | ECG | binary |  | QRS duration greater than or equal to 120 ms in adults; rsr’, rsR’, or rSR’ in leads V1 or V2. The R’ or r’ deflection is usually wider than the initial R wave. In a minority of patients, a wide and often notched R wave pattern may be seen in lead V1 and/or V2; S wave of greater duration than R wave or greater than 40 ms in leads I and V6 in adults | 0 |
| Incomplete RBBB | ECG | binary |  | QRS duration between 110 and 120 ms. rsr’, rsR’, or rSR’ in leads V1 or V2. | 0 |
| Non-specific intraventricular delay | ECG | binary |  | QRS duration greater than 110 ms without criteria for RBBB or LBBB; The definition may also be applied to a pattern with RBBB criteria in the precordial leads and LBBB criteria in the limb leads, and vice versa | 0 |
| LAFB | ECG | binary |  | Frontal plane axis between -45° and -90°; qR pattern in lead aVL; R-peak time in lead aVL of 45 ms or more; QRS duration less than 120 ms | 0 |
| Tr V1 V2 | ECG | binary |  | QRS transition between leads V1 and V2 | 0 |
| Tr V2 V3 | ECG | binary |  | QRS transition between leads V2 and V3 | 0 |
| Tr V3 V4 | ECG | binary |  | QRS transition between leads V3 and V4 | 0 |
| Tr V4 V5 | ECG | binary |  | QRS transition between leads V4 and V5 | 0 |
| Tr V5 V6 | ECG | binary |  | QRS transition between leads V5 and V6 | 0 |
| SV1 | ECG |  | mm | Voltage amplitude of S wave in V1 | 0 |
| SV2 | ECG |  | mm | Voltage amplitude of S wave in V2 | 0 |
| SV3 | ECG |  | mm | Voltage amplitude of S wave in V3 | 0 |
| SV4 | ECG |  | mm | Voltage amplitude of S wave in V4 | 0 |
| RV5 | ECG |  | mm | Voltage amplitude of R wave in V5 | 0 |
| RV6 | ECG |  | mm | Voltage amplitude of R wave in V6 | 0 |
| RDI | ECG |  | mm | Voltage amplitude of R wave in DI | 0 |
| RDII | ECG |  | mm | Voltage amplitude of R wave in DII | 0 |
| SDII | ECG |  | mm | Voltage amplitude of S wave in DII | 0 |
| SDIII | ECG |  | mm | Voltage amplitude of S wave in DIII | 0 |
| SaVR | ECG |  | mm | Voltage amplitude of S wave in aVR | 0 |
| RaVL | ECG |  | mm | Voltage amplitude of R wave in aVL | 0 |
| RaVF | ECG |  | mm | Voltage amplitude of R wave in aVF | 0 |
| Intrinsicoid deflection | ECG | binary |  | Amplitude of the intrinsicoid deflection in V5 or V6 > 50 msec in the lead exhibiting QRS transition (the point at which the QRS complex changes from predominantly negative to mostly positive RS pattern) | 0 |
| secondary ST-T segment abnormalities | ECG | binary |  | secondary ST-T segment abnormalities | 0 |
| LVH for Sokolow-Lyon criteria | ECG | binary |  | sum of S wave in V1 and R wave in V5 or V6 ≥3.5 mV (35 mm) and/or R wave in aVL ≥1.1 mV (11 mm) | 0 |
| LVH for Cornell criteria | ECG | binary |  | For men: S in V3 plus R in aVL >2.8 mV (28 mm); for women: S in V3 + R in aVL >2.0 mV (20 mm) | 0 |
| Low voltages (peripheral) | ECG | binary |  | QRS complex voltage (peak-to-peak) < 5 mm in all peripheral leads simultaneously. | 0 |
| Low voltages (precordial) | ECG | binary |  | QRS complex voltage < 10 mm in all precordial leads simultaneously. | 0 |
| Low voltages (total) | ECG | binary |  | simultaneous presence of low voltages in peripheral and precordial leads | 0 |
| S nadir | ECG |  | ms | duration from the nadir of the S wave to its end (in V1 or V2) | 0 |
| Early repolarization | ECG | binary |  | - J point elevation > 0.1 mV at least in two leads - J point elevation > 0.2 mV at least in two leads - Localization: inferior (DII, DIII, aVF), lateral (DI, aVL, V4,V5,V6) o V1-V3 - Morphology: notched, slurred, mixed - ST Morphology: ascending, horizontal, mixed | 0 |
| fQRS | ECG | binary |  | fragmented QRS defined as additional R wave (R’), notching of the R wave or of the S wave; Presence of > 1 R’ in two contiguous leads in patients with narrow QRS (QRS < 120 ms) | 0 |
| T wave duration | ECG |  | ms | T wave duration | 0 |
| T wave amplitude | ECG |  | mm | T wave amplitude | 0 |
| Nadir-to-end T wave | ECG |  | mm | in V5 | 0 |
| Inverted T wave in DI | ECG | binary |  | Inverted T wave in DI | 0 |
| Inverted T wave in DII | ECG | binary |  | Inverted T wave in DII | 0 |
| Inverted T wave in DIII | ECG | binary |  | Inverted T wave in DIII | 0 |
| Inverted T wave in aVF | ECG | binary |  | Inverted T wave in aVF | 0 |
| Inverted T wave in aVR | ECG | binary |  | Inverted T wave in aVR | 0 |
| Inverted T wave in aVL | ECG | binary |  | Inverted T wave in aVL | 0 |
| Inverted T wave in V1 | ECG | binary |  | Inverted T wave in V1 | 0 |
| Inverted T wave in V2 | ECG | binary |  | Inverted T wave in V2 | 0 |
| Inverted T wave in V3 | ECG | binary |  | Inverted T wave in V3 | 0 |
| Inverted T wave in V4 | ECG | binary |  | Inverted T wave in V4 | 0 |
| Inverted T wave in V5 | ECG | binary |  | Inverted T wave in V5 | 0 |
| Inverted T wave in V6 | ECG | binary |  | Inverted T wave in V6 | 0 |
| Inverted T wave in infero-lateral leads | ECG | binary |  | Inverted T wave in infero-lateral leads | 0 |
| Inverted T wave in antero-lateral leads | ECG | binary |  | Inverted T wave in antero-lateral leads | 0 |
| NYHA 3-4 |  | binary |  | NYHA 3-4 | 0 |
| Right ventricle systolic disfunction | Echocardiogram | binary |  | Fractional area change<35% | 1 |
| RV1>0 | ECG | binary |  | positive QRS deflection in V1 | 0 |
| RV2>0 | ECG | binary |  | positive QRS deflection in V2 | 0 |
| RV3>0 | ECG | binary |  | positive QRS deflection in V3 | 0 |
| RV4>0 | ECG | binary |  | positive QRS deflection in V4 | 0 |
| SV5>0 | ECG | binary |  | negative QRS deflection in V5 | 0 |
| SV6>0 | ECG | binary |  | negative QRS deflection in V6 | 0 |
| SDI>0 | ECG | binary |  | negative QRS deflection in dI | 0 |
| RDIII>0 | ECG | binary |  | positive QRS deflection in dIII | 0 |
| RaVR>0 | ECG | binary |  | positive QRS deflection in aVR | 0 |

**Supplementary Table S2**. Additional baseline characteristics of the study cohort.

* missing data in 1 individual.

† missing data in 5 individuals.

|  | **Study cohort**  **N=409** |
| --- | --- |
| Syncope  N (%) | 34 (8) |
| NYHA  N (%) |  |
| 1 | 207 (51) |
| 2 | 121 (30) |
| 3 | 59 (14) |
| 4 | 22 (5) |
| Family history of CMP  N (%) | 139 (34)* |
| Family history of SCD  N (%) | 62 (15)† |
| Atrial fibrillation  N (%) | 14 (3) |
| ‍Non-Dilated Left Ventricular Cardiomyopathy | 82 (20%)† |

**Supplementary Table S3.** Multivariable analysis for SCD/MVA events after excluding individuals with non-dilated left ventricular cardiomyopathy (81 individuals).

| **Variable** | HR | 95% CI | | p-value |
| --- | --- | --- | --- | --- |
| Sex | 1.75 | ‍0.93 | 3.32 | ‍0.085 |
| Age | 1.01 | ‍0.99 | 1.03‍ | ‍0.3 |
| Family history of SCD | 1.30 | ‍0.67 | 2.52‍ | ‍0.4 |
| Inverted T wave in infero-lateral leads | 1.58 | ‍0.87 | 2.85 | ‍0.13 |
| **Syncope** | 3.60 | ‍1.89 | 6.86 | ‍<0.001 |
| NYHA 3-4 | 1.44 | ‍0.78 | 2.65 | ‍0.2 |
| LVEF | 0.99 | ‍0.96 | 1.01‍ | ‍0.4 |
| **CL2** | 0.15 | ‍0.05 | 0.44 | ‍<0.001 |

**Supplementary Table S4.** Regression coefficients of the LASSO penalized model. *Example*: a patient with V6 QRS interval duration 110ms, presence of true LBBB and absence of intrinsicoid deflection obtains a score of 1/(1+exp(110*0.0054+1*2.6086+0*1.1659))=0.0391. Since this value is <0.23, the subject is assigned to CL1.

| **Variable** | **Beta** |
| --- | --- |
| ‍V6 QRS interval duration | 0.0054 |
| ‍True LBBB | 2.6086 |
| Intrinsicoid deflection | 1.1659 |
