# Supplementary figures and images for: Clustering in dilated cardiomyopathy at initial evaluation: An effective tool for clinical stratification
Source: Eur J Heart Fail. 2025 Aug 15;27(12):3040–7. doi: 10.1002/ejhf.3780 (PMC12803628; doi:10.1002/ejhf.3780)

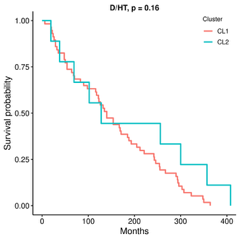

Supplement: Supplementary file 2 — Figure S1 Death and heart transplant survival curve. Kaplan–Meier curve for D/HT events in CL1 and CL2 [file EJHF-27-3040-s001.tiff]

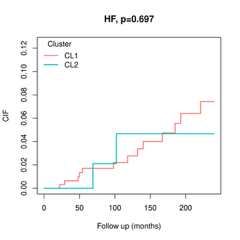

Supplement: Supplementary file 3 — Figure S2 Heart failure in the two clusters. Cumulative incidence functions for HF events in CL1 and CL2. [file EJHF-27-3040-s003.tiff]

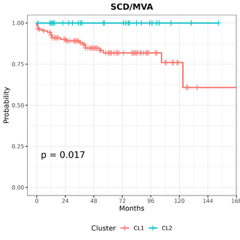

Supplement: Supplementary file 4 — Figure S3 Risk of severe arrhythmic event in the validation cohort. Comparison of Kaplan–Meier curves for SCD/MVA events between CL1 and CL2 in the validation cohort. [file EJHF-27-3040-s002.tiff]
